# Local Anesthesia

**Published:** 1901-09

**Authors:** F. C. Floeckinger

**Affiliations:** La Grange, Texas


### Local Anesthesia.

By F. C. FLOECKINGER, M. D., La Grange, Texas.

LOCAL anesthesia has incontestably conquered the field in surgical practice. The discoverer of this method, Corning, of New York, (New York *Medical Journal*, 1887, Vol. XLII., No. 12,) found by experience that after constriction of a limb and injection of a 4 per cent. solution of cocain muriate in the vicinity of a nerve trunk, peripherally from the constricting-coil, complete anesthesia supervened after the expiration of a few minutes.

Corning, however, applied this method only to the external cutaneous nerve of the arm, and his experiment fell into oblivion until Oberst of the Surgical Clinic, at Halle, undertook further experiments and published his results. Oberst demonstrated that it is quite unnecessary to employ a greater strength of cocain than a 1 per cent. solution, and he maintained that the analgesic effect is due chiefly to the pressure of the constricting-coil, and the resulting complete interruption of the circulation—*i.e.*, artificial ischemia. Pernice published Oberst's experience in the *Deutsche Med. Wochen.*, 1890, No. 14.

Among the physicians who have been conspicuous for their services in this field, Schleich, H. Braun and Otto Maur deserve especial mention; but it would overstep the limits of my space to give all that has been said for and against this method by the physicians who have occupied themselves therewith.

The method extends to the extremities and penis, or, in other words, to all portions of the body which may be rendered ischemic by constriction. It is astonishing how little country practitioners have occupied themselves with this method, when the latter is so simple, and, when a 1 per cent. or 2 per cent. solution of nirvanin is used, so completely devoid of danger. Some colleagues, perhaps, have made a trial of this method without securing the desired result; but in these cases the blame is to be attributed to defective technique. One must learn to use this method, and I leave it for the clinicians to show if my words are not correct. That it is also necessary for the physician to have the anatomy of the nerves in his head, hardly needs to be mentioned; and a few trials combined with patience will be crowned with success in a short time. I have demonstrated these methods to several of my colleagues, and they have marveled at the facility with which good results are obtained.

When the subject of local anesthesia was in its initial stages, cocain was used in strengths of 4 per cent., 3 per cent., and 2 per cent., and cases occur in literature in which the anesthesia was accompanied by toxic phenomena. For this reason, many physicians have feared to apply this method. Later eucain was introduced into practice and used in many clinics. Wohlgemuth, in a discussion before the 27th Congress of the Deutsche Gesellschaft für Chirurgie, stated that eucain possessed no advantage over cocain, and did not produce as good anesthesia as the latter. All these disadvantages were obviated by the introduction into practice of nirvanin. My space does not permit me to go more closely into the physiological action of this preparation, and I, therefore, refer the reader to the literature which has appeared on this subject, and I shall only remark here that nirvanin is ten times less toxic than cocain, and further, shows antiseptic qualities, with the result that solutions of it may be preserved for a long period without spoiling.

The operations which I have performed under anesthesia by nirvanin injections by the aid of the Oberst-Braun method, since the publication of my first article are as follows: 28 cases of panaritium of mixed nature; 2 cases of lipoma of the extremities; 1 case of osteomyelitis with necrosis of the tibia; 1 case multiple ligation of the branches of the greater and lesser saphenous veins; 1 case of disarticulation of the thumb at the metacarpo-phalangeal joint; 6 cases of plegmon of the palm of the hand—septic nature with abscess formation beneath the deep palmar fascia; 4 cases of ingrown nail; 4 cases of circumcision.

I will here discuss the cases so far as may be necessary without giving case-histories, for the latter would transcend the space which stands at my disposal.

In ordinary cases I apply a 2 per cent. solution of nirvanin, and in extensive operations, in which a large amount of injection-fluid must be used, 1 per cent. and ½ per cent. solutions suffice. As a solvent for nirvanin, I employ a decinormal salt solution with addition of a little muriate of morphia to lessen the pain of the injection.

In order to open felons painlessly, I make use of the following method: The finger concerned is encircled with two turns of a thin rubber tube, one inch from the site of the median-lateral incision. After waiting a few moments a second rubber tube is applied behind the first one, which latter is then removed. The second tube produces a greater degree of constriction than the first. I employ this method in all cases because I have observed that to constrict too tightly with the first tube causes too severe pain. In general the patient immediately complains after the constriction is applied of marked pressure pain, but this is abated in a few moments. After the constriction of the limb, a syringe holding 10 c.c. with a fine nozzle, having a sharp point, is filled with a 2 per cent. solution of nirvanin, and the injection is made laterally in the aponeurosis of the finger. In order to render the first puncture painless I employ the chlorid of ethyl spray. The needle is introduced distally from the constricting tube in a peripheral direction, and the fluid slowly injected. Quite an amount of pressure is necessary in order to empty the syringe. After practicing the injection on one side of the finger the process is repeated on the other side and the operator waits for a few moments. It is difficult to state the exact interval of delay necessary before operating, as this depends upon the degree of inflammation, and in one case I was obliged to wait twenty-three minutes before I could carry out a painless incision. Patients often complain for hours over persistent pains at the place of constriction.

One portion of the modus operandi is of importance; this is, the application of the constricting tube which should occur as far peripherally as possible, as thereby the anesthesia will appear sooner and last longer. On an average I was successful with one grain of nirvanin, and in no case did I observe toxic phenomena. The closer the rubber was applied peripherally, the less injection fluid was required. If the constriction was applied at the base of the finger, four punctures were necessary on the average, viz.: two dorsal and two palmar. The needle should not enter too deeply in order to better encounter the direct surroundings of the nerve, for the latter lie upon the dorsal and palmar aponeuroses. In my first attempts in this field, I introduced the needle as deep as the periosteum, and much more time was required before analgesia set in, while the latter was not complete. However, it is self evident that after extinction of the sensory activity of the main trunk the function of the peripheral branches is also lost.

In both my cases of extirpation of lipoma nothing occurred worthy of mention, save that both growths developed after trauma. The latter consisted of a blow without solution of continuity of the skin. One tumor was seated on the lower extremity, two inches below the point of passage of the superficial peroneal nerve, and had the circumference of a silver dollar. The first constriction of the leg was applied one inch above the tumor, and the second was applied in front of the first. The first was then removed and a single injection of 2 per cent solution of nirvanin produced complete anesthesia of the integument. A second syringe was emptied at the base of the tumor, and after waiting four minutes the operation could be carried out painlessly.

The other tumor was seated on the volar surface of the forearm, somewhat peripherally before the point of exit of the palmar cutaneous branch of the median nerve. Its excision was painless.

The opening of the abscess of bone in the tibia offers certain interesting points. Constriction of the limb was practised below the knee joint, and in all there were injected 50 c.cm. of a 1 per cent. solution of nirvanin. Injection was made in the vicinity of the greater saphenous, deep peroneal and superficial peroneal —before this breaks through the aponeurosis—directly before the constricting coil. The incision in the tissues was wholly devoid of sensation, and only the penetration of the probe into the carious bony cavity was somewhat painful. I injected a few drops of a 5 per cent. solution into the inflamed periosteum after previously treating the site of the puncture with a 20 per cent. solution. After waiting a few minutes I could carry out the chiseling of the bone with a complete absence of pain. The scooping out of the bony cavity with a sharp spoon was also completely painless.

In ligating the branches of the vena saphena major and minor, in the leg, I injected directly into the vicinity of the field of operation, as this lay directly below the constriction coil. The greater saphenous nerve and the tibial nerve run, after their passage through the aponeurosis, directly with the major and minor saphenous veins, and extirpation of a piece of vein, an inch or so in length, could be carried out in perfectly painless fashion.

In disarticulation of the thumb four injections were made at the base of the thumb, and one injection directly among the ligaments which form the joint. After nine minutes delay the operation could be performed in a perfectly painless manner.

The opening of the abscesses in the palm of the hand required a good deal of injection fluid, so that the strength of solution used was but ½ per cent. Constriction was made at the wrist, and the needle introduced into the deep tissues, so that the deeper seated nerve filaments could be rendered analgesic.

It is true that injection into the deeper tissues requires some force, though I could obtain no advantage from the pumping apparatus used by Prof. Matas, at the New Orleans Polyclinic. For a clinician who has to operate several times in this manner in rapid succession, this method may be well to use, but the country practitioner must know how to help himself by the simplest methods.

The method of injection in operating upon ingrown nails offers nothing special for discussion; it is only necessary to inject one syringe full beneath the nail and into the nailbed.

In operating for circumcision the constricting coil was applied directly above the sulcus coronarius, and the injection made directly into the tissues of the penis. By this means both lamina of the prepuce are made insensitive. All operations may be made painless by the use of the average of 2 per cent. nirvanin solution.

In conclusion, I will briefly remark that this method gives excellent results if the technique is under control, and the operator has sufficient patience.

LITERATURE.

Braun (Leipzig), Centralblatt für Chirurgie, 1897, No. 17.
Ruge, Charité Annalen, 1896.
Otto Manz (Frieburg), Centralblatt für Chirurgie, 1898, No. 7.
Paul Arndt (Antwerp), Centralblatt für Chirurgie, 1898, No. 16.
Schleich, Schmerzlose Operationen, Berlin, 1898.
Briegleb (Worms), Zeitsch. für Prak. Aerzte, 1897, No. 9.
Briegleb (Worms), Kinderarzt, 1897, No. 11 and 12.
Rubinstein (Berlin), 27th Congress der Deutschen Gesellschaft für Chirurgie.
Wohlgemut (Berlin), Discussion.
Mankiesvics (Berlin), Discussion.
Gottstein (Breslau), Discussion.
Reclus La Cocain in Chirurgie, 1895.
Sudek, Deutsche med. Wochenschrift, 1898, No. 8.
Krieshaber, Gaz. med. de Paris, 1874, p. 293, 307.
Vernenil, Gaz. de Hospitaux, 1874, No. 103.
Neuber, Utersuchungen und Erfahrungen über künstliche Blutleere, Disc., Kiel. 1878.
Iverson, Künstliche Ischemie bei Operationen, Kiel, 1873.
Le Fort, Gaz. de Hospitaux, 1874, No. 103.
Stokes, Dublin Press and Circular, 1874, p. 248.
Karenski, Therapeutische Monatshefte, 1888, p. 168.
Floeckingar, American Therapist, July, 1900.
Corning, New York Med. Journal, 1887, Vol. 42, No. 12.

---

RIESMAN (*American Journal of the Medical Sciences*, July, 1901,) in an interesting description of a case of primary tubercular pericarditis, emphasises the fact that cases of adherent pericardium ascites is usually noticed before the appearance of edema of the lower extremities.

